# The Involvement of *Mycobacterium* Type III-A CRISPR-Cas System in Oxidative Stress

**DOI:** 10.3389/fmicb.2021.774492

**Published:** 2021-12-09

**Authors:** Fan Yang, Lingqing Xu, Lujie Liang, Wanfei Liang, Jiachen Li, Daixi Lin, Min Dai, Dianrong Zhou, Yaxin Li, Yong Chen, Hui Zhao, Guo-bao Tian, Siyuan Feng

**Affiliations:** ^1^Department of Microbiology, School of Basic Medical Science, Xinxiang Medical University, Xinxiang, China; ^2^Department of Clinical Laboratory, The Sixth Affiliated Hospital of Guangzhou Medical University, Qingyuan People’s Hospital, Qingyuan, China; ^3^Department of Microbiology, Zhongshan School of Medicine, Sun Yat-sen University, Guangzhou, China; ^4^Key Laboratory of Tropical Diseases Control (Sun Yat-sen University), Ministry of Education, Guangzhou, China; ^5^School of Laboratory Medicine, Chengdu Medical College, Chengdu, China; ^6^Guangdong Provincial Key Laboratory of Biotechnology for Plant Development, School of Life Sciences, South China Normal University, Guangzhou, China

**Keywords:** type III-A CRISPR-Cas, *Mycobacterium*, envelope integrity, oxidative stress, immunoregulation

## Abstract

Type I and type II CRISPR-Cas systems are employed to evade host immunity by targeting interference of bacteria’s own genes. Although *Mycobacterium tuberculosis* (*M. tuberculosis*), the causative agent of tuberculosis, possesses integrated type III-A CRISPR-Cas system, its role in mycobacteria remains obscure. Here, we observed that seven cas genes (*csm2*∼*5*, *cas10*, *cas6*) were upregulated in *Mycobacterium bovis* BCG under oxidative stress treatment, indicating the role of type III-A CRISPR-Cas system in oxidative stress. To explore the functional role of type III-A CRISPR-Cas system, TCC (*T*ype III-A *C*RISPR*-C*as system, including *cas6*, *cas10*, and *csm2*-*6*) mutant was generated. Deletion of TCC results in increased sensitivity in response to hydrogen peroxide and reduced cell envelope integrity. Analysis of RNA-seq dataset revealed that TCC impacted on the oxidation-reduction process and the composition of cell wall which is essential for mycobacterial envelop integrity. Moreover, disrupting TCC led to poor intracellular survival *in vivo* and *in vitro*. Finally, we showed for the first time that TCC contributed to the regulation of regulatory T cell population, supporting a role of TCC in modulating host immunity. Our finding reveals the important role of TCC in cell envelop homeostasis. Our work also highlights type III-A CRISPR-Cas system as an important factor for intracellular survival and host immunoregulation in mycobacteria, thus may be a potential target for therapy.

## Introduction

CRISPR-Cas (Clustered regularly interspaced short palindromic repeats-CRISPR associated) are found in approximately 50% of sequenced bacterial genomes and nearly 90% of sequenced archaea (Hille et al., 2018). Based on the architectures of effector modules, the CRISPR-Cas systems can be divided into two major classes. The class 1 CRISPR-Cas systems consist of large, multisubunit Cas protein complexes, whereas class 2 CRISPR-Cas systems perform the function by a single large protein. These systems provide adaptive immunity against foreign nucleic acids. In addition to their role in bacterial immunity, increasing evidence reveals that the CRISPR-Cas systems impact bacterial physiology and virulence. For example, *Francisella novicida* employ type II CRISPR/Cas to dampen a proinflammatory innate immune response by repressing transcription of a bacterial lipoprotein (Sampson et al., 2013), which suppresses TLR2-dependent proinflammatory responses in macrophages (Jones et al., 2012). *Pseudomonas aeruginosa* type I CRISPR-Cas system dampen the recognition by toll-like receptor 4 via targeting the mRNA of the bacterial quorum-sensing regulator *lasR* (Li et al., 2016). These findings support the importance of CRISPR-Cas system in bacterial pathogenesis.

It has been shown that type III CRISPR-Cas system belongs to class 1 CRISPR-Cas systems and contains diverse and polymorphic CRISPR-Cas loci with high divergence between the subtypes (Makarova et al., 2015), such as Csm (Type III-A) complexes and Cmr (Type III-B) complexes. Well-established type III CRISPR-Cas system have a unique targeting mechanism that is able to degrade both the DNA and RNA of invader (Pyenson and Marraffini, 2017). For example, mature crRNA was generated by the Cas6 endonuclease, the complementary target RNA was recognized by crRNA-guided Csm/Cmr complexes and then Csm3 or Cmr4 subunit cleave target RNA into 6 nt nucleotide intervals, while the DNA cleavage was catalyzed by the single-stranded DNase activity of Cas10 (Samai et al., 2015; Park et al., 2017; Tamulaitis et al., 2017). Once Cas10 degraded DNA, cleavage of the protospacer RNA by Csm6/1 resulted in inactivation of Cas10. In addition, the Palm domain of Cas10 synthesized cyclic oligoadenylate (cOA), which was required for modulating the endoribonuclease activity of Csm6 (Rostol and Marraffini, 2019). These systems can broadly eliminate foreign targets with multiple mutations but circumvent damage to the host genome. Although recent studies have shown that the molecular mechanism of type III CRISPR-Cas systems against exogenous DNA, it is unclear whether type III CRISPR-Cas system is associated with bacterial physiology to date.

*Mycobacterium tuberculosis* is one of the leading causes of death from an infectious disease. Integrated type III-A CRISPR-Cas system is predominantly presented in the slow-growing mycobacteria, such as *M. tuberculosis* and *M. bovis* (Singh et al., 2021), rather than non-pathogenic mycobacteria (He et al., 2012). In the virulent *M. tuberculosis* strain H37Rv, the type III-A system is comprised of Csm2-6, Cas10, Cas6, Cas1, Cas2, and two CRISPR loci. In addition to its role in *M. tuberculosis* strain genotyping, recent studies have focused on the activity of *M. tuberculosis* CRISPR interference system in invader defense and potential for an endogenous genome editing tool.

A recent study demonstrated that deletion of *cpnB*, which encodes a multifunctional protein that hydrolyzes c-di-AMP, c-di-GMP, and nanoRNAs, upregulated all the cas (CRISPR-associated) genes compared to the WT (Zhang et al., 2018). Of note, the expression of cas gene varies in response to environmental stresses, such as oxidative stress and antibiotic treatment (Miryala et al., 2019), suggesting a link between the activity of type III-A CRISPR-Cas and stresses. Indeed, it has been reported that overexpression of cas genes impacts mycobacterial physiology (Huang et al., 2016; Zhai et al., 2018; Wei et al., 2019). For example, overexpression of *cas2* decreased the survival ability of *Mycobacterium smegmatis* under oxidative stress (Huang et al., 2016). Cas1 impaired the repair of DNA damage and reduced the ability of *M. smegmatis* to resist oxidative stress. Although these studies indicated that a role for type III-A CRISPR-Cas in adapting to stressful environments in fast-growing mycobacteria, the function of type III-A CRISPR-Cas system in slowly growing mycobacteria is unclear.

The current study aimed to explore the role of type III-A CRISPR-Cas system in slowly growing mycobacteria. By studying *Mycobacterium bovis* BCG, we found that type III-A CRISPR-Cas system is required for adaptation to oxidative stress and cell envelope integrity. We also proved the involvement of type III-A CRISPR in regulating gene expression by RNA-seq. As expected, intracellular fitness significantly declines due to loss of type III-A CRISPR system. Moreover, mice infected with ΔTCC (type III-A CRISPR system mutant) increased the regulatory T cell population compared to WT control. This study could shed light on the molecular mechanisms of type III-A CRISPR system in mycobacteria.

## Materials and Methods

### Strains and Media

*Mycobacterium bovis* BCG and *M. smegmatis* MC^2^ 155 were grown at 37°C in Middlebrook 7H9 broth (BD Biosciences), 7H10 agar plates (BD Biosciences) supplemented with 0.5% glycerol, 0.05% Tween-80, 10% oleic acid ADC and appropriate antibiotics. When indicated, antibiotics and inducers were used at the following concentrations: kanamycin (20 μg/ml, Sangon Biotech), hygromycin (50 μg/ml, Sangon Biotech), and anhydrotetracycline (ATc; 50 ng/ml, Abcam). The *Escherichia coli* strain DH5α was used for plasmid cloning and was cultivated at 37°C in Luria-Bertani (LB) broth or agar plates.

### Ethics Statement

All animal experiments were performed in accordance with the National Institutes of Health Guide for the Care and Use of Laboratory Animals, and the experimental procedures were approved by the Ethics Committee of Zhongshan School of Medicine on Laboratory Animal Care (reference number: 2016-159), Sun Yat-sen University.

### Strain Construction

To generate ΔTCC, or *cas6* knock-out in *M. bovis* BCG strain, deletion strain was generated by phage specialized transduction as described previously (Jain et al., 2014). Briefly, PCR was performed to synthesize fragments bearing the ∼1,000 and ∼1,000 bps of flanking regions of endogenous gene of *M. bovis* BCG, resulting in a deletion of the gene. The primers used in this study are listed in Supplementary Table 1. Amplicons corresponding to upstream and downstream flanking regions were digested with *Van*91I and cloned into the *Van*91I (Thermo Fisher Scientific) digested p0004s plasmid that contains a hygromycin resistance cassette and the *sacB* gene to be able to select for sucrose sensitivity. This allelic exchange substrate was introduced into the *Pac*I (Thermo Fisher Scientific) site of phasmid phAE159 and electroporated into *M. smegmatis* mc^2^155 to obtain high titers of phage phAE159. Subsequently, the *M. bovis* BCG wild-type strain was incubated with high titers of the corresponding phage to create TCC, or *cas6* knockouts, respectively. Colonies that had deleted endogenous TCC, or *cas6* were selected on hygromycin-containing plates and verified for sucrose sensitivity, respectively. The deletion was confirmed by PCR analysis and sequencing (Supplementary Figure 2).

For construction of the complementation strain, TCC with its native promoter was first cloned into a pMV306 (mycobacterial integration vector; integrates into the attB site; Kan*^R^*), and the recombinant plasmid pMV306-TCC was integrated into the chromosomes of the deletion strains, resulting in *comp* strain. The comp strain was selected in Middlebrook 7H10 agar medium (complemented with 10% OADC) containing 20 μg/ml kanamycin.

### Construction of CRISPR Interference (CRISPRi) Targeting Constructs in *M. bovis* BCG

CRISPRi targeting constructs in *M. bovis* BCG was performed as previously described (Rock et al., 2017). Briefly, we designed sgRNA to target the non-target strand of *whiB6* and *bcg_1807* gene. For each individual sgRNA, two complementary oligonucleotides with appropriate sticky end overhangs were annealed to generate a double-stranded insertion which was phosphorylated by T4 polynucleotide kinase (Thermo Fisher Scientific). PCR conditions were: 37°C for 30 min, 95°C for 5 min, ramp down to 25°C at 5°C min^–1^. And then PCR products was ligated (T4 DNA ligase, Thermo Fisher Scientific) into the BsmBI-v2 (NEB) digested pLJR965 to obtain pLJR965-sgRNA. Successful cloning was confirmed by Sanger sequencing. pLJR965 containing sgRNA-targeting the genes of interest was transformed into *M. bovis* BCG competent cells. For each transformation, 100 ng plasmid and 100 μl pf electrocompetent mycobacteria were used. Transformants were selected by plating cells on 7H10-OADC plates containing 50 μg/ml hygromycin and 20 μg/ml kanamycin. A single-clone was picked and cultivated to log phase in the absence of ATc and then diluted back to OD_600_ = 0.015 with or without 100 ng/ml ATc. After 10 days of outgrowth, 0.4 OD_600_ units of cells was harvested by centrifugation and processed for RNA extraction.

### Analysis of *in vitro* Response to Oxidative Stress

Exponentially growing bacterial cultures (OD_600_ 0.4–0.6) were pelleted and washed three times with 7H9 containing 0.05% Tween-80, then diluted to OD_600_ = 0.5. The cells were treated only in 7H9 broth medium with different amounts of H_2_O_2_ (0, 5, 10, and 50 mM) and were incubated for 24 h. Then OD_600_ were recorded by using Nano-photometer (IMPLEN) and normalized to the corresponding control without oxidant treatment. When indicated, bacteria strains were serially diluted ten-fold with PBS immediately and spotted on 7H10-OADC agar. CFUs of *M. bovis* were counted after 4 weeks of incubation at 37°C.

### Synthesis of cDNA and RT-PCR

Exponentially growing bacterial cultures (OD_600_ 0.4–0.6) were pelleted and washed once with 7H9 containing 0.05% Tween-80, then diluted to OD_600_ = 0.5. The cells were treated with different stresses, including 5 mM H_2_O_2_, 5 mM NO, acidic medium (pH4.5), 1%SDS and were incubated for 2 h. Equal amounts of bacteria of *M. bovis* strains were collected. Total RNA and cDNA from *M. bovis* BCG were extracted as previously described (Rustad et al., 2009). Briefly, the collected cultures were harvested by centrifugation. After removal of the supernatant, the cell pellet was resuspended in RNA-easy Isolation Reagent (Vazyme) and disrupted by bead beating with a TissueLyser-II (QIAGEN). Total RNA was precipitated by the addition of isopropanol and collected by centrifugation, the supernatant was discarded, and the mRNA pellet was washed with 75% ethanol. After drying of the pellet, mRNA was dissolved in RNase-free H_2_O. The contaminating genomic DNA was digested with gDNA wiper Mix (Vazyme). The cDNA was prepared from purified mRNA with HiScriptII qRT SuperMix II (Vazyme) through reverse transcription. The cDNA levels of target genes were then quantified by quantitative real-time PCR (qRT-PCR) on a CFX96 cycler (BIO RAD) by using AceQ Universal SYBR qPCR Master Mix (Vazyme) as a dye for fluorescence signal detection. All qPCR primers were determined to be >95% efficient, and the cDNA masses tested were experimentally validated to be within the linear dynamic range of the assay. Signals were normalized to those of the housekeeping *sigA* transcript and quantified with the ΔΔ*C*t method. Error bars are 95% confidence intervals of the three technical replicates.

### Growth Curves

*Mycobacterium bovis* strains were grown in 7H9 medium containing OADC-Tween 80 to mid-log phase (OD_600_, 0.5–0.8). The precultures were pelleted at 3,000 g for 10 min and washed once in PBS, then diluted to OD 0.1 in 7H9 medium containing OADC-Tween 80. A Nano-photometer (IMPLEN) was used to measure the optical density at indicated time. The growth curve was generated based on the OD_600_. This experiment was performed in three replicates.

### *In vitro* Permeability

Mycobacteria strains were grown to an OD_600_ 0.5–1.0. bacteria were collected by centrifugation at 8,000 *g* for 10 min at 4°C. The collected cells were further washed three times using 1× PBS buffer then suspended in 1× PBST containing 50 μg/ml EtBr (Sangon Biotech) or 20 μM Nile Red (Sangon Biotech). Fluorescence was measured immediately in a Spectra Max M5 (Molecular Devices) using an excitation of 540 nm and emission of 590 nm, correcting with samples lacking bacteria. All experiments were repeated in triplicate.

### RNA Preparation and RNA-Seq Analysis

RNA preparation and RNA-seq analysis were performed as previously described (Feng et al., 2020). Briefly, wild-type *M. bovis* BCG as well as the TCC deletion strain were grown to the log-phase (OD_600_, 0.5) in Middlebrook 7H9-OADC. Total RNA was prepared from wild-type and mutant strains using TRIzol reagent (Invitrogen), followed by DNase I treatment. Approximately 1-μg total RNA samples were treated by the Ribo-Zero rRNA removal procedure (Illumina) to enrichment for mRNA. Approximately 1 μg of RNA was used for library preparation using a ScriptSeq (v2) RNA-seq kit and high-throughput sequencing on an Illumina NextSeq platform. All raw sequence reads by RNA-Seq were initially pre-processed by Trimmomatic (v0.36) to trim the adaptor sequences and remove low-quality sequences. The remaining clean reads were mapped to the *M. bovis* BCG genomes using Tophat2. The alignment results were input into Cufflinks (v2.2.1). Unless otherwise stated, the unit of expression level in our analyses is FPKM. Cuffdiff (v2.2.1) was used to test for differential expression. The genome sequences and the annotations of *M. bovis* BCG were obtained from the GenBank^1^. The genes with a *p*-value ≤ 0.05 and |log_2_ fold change (ΔTCC/WT)| ≥ 1 were identified as differentially expression genes. The data for visualization was generated by R (R Development Core Team, Vienna, Austria). The functional annotations of differentially expression genes were carried out by gene ontology (GO) from an internationally established system (GO^2^). This system comprehensively describes the genes’ properties and provides their products in any organism. The corrected *p*-value of GO terms (*p* < 0.05) was considered significantly enriched by differentially expression genes. Differentially expression genes analyses were performed on the basis of the biological process, molecular function, and cellular components. The Kyoto Encyclopedia of Genes and Genomes (KEGG) database was used to determine the molecular pathways for the differentially expression genes.

### Biofilm Growth Conditions

Mycobacteria strains were cultured in Sauton’s medium without Tween 80 in 12-well polystyrene tissue culture plates (Corning). Plates were incubated at 37°C under 5% CO_2_ for 5 weeks.

### Cell Culture

Murine macrophage-like RAW264.7 cells (ATCC; TIB-71) were cultured on Dulbecco’s Modified Eagle Medium (DMEM, Invitrogen) supplemented with 10% fetal bovine serum (FBS), 100 U/ml penicillin, and 100 μg/ml streptomycin (GIBCO, Invitrogen) with 5% CO_2_ at 37°C.

### Macrophage Infection Study

RAW264.7 cells were seeded at 3 × 10^5^ cells/well in a 24-well plate and incubated for 18 h. Bacterial cells were expanded to OD_600_ = 1, pooled and passed through a 10 μm cell strainer to obtain a single cell suspension. Adherent RAW264.7 cells were infected with homogenized bacterial suspension at a multiplicity of infection (MOI) of 10. The extracellular bacteria were removed by washing three times with phosphate buffer saline (PBS) after incubation for 2 h. Cells were further incubated for 48 h in a CO_2_ incubator at 37°C. Then cell lysates were serially diluted 10-fold with PBS immediately and spotted on 7H10-OADC agar. CFUs of *M. bovis* were counted after 4 weeks of incubation at 37°C.

### Animal Experimentation

Female C57BL/6 mice were purchased from Animal Supply Center, Sun Yat-sen University. Mice were bred and maintained under specific pathogen-free conditions at the animal facility of the Sun Yat-sen University. Adult mice between 6 and 8 weeks of age were used, and infected mice were maintained under biosafety 2 conditions. To induce acute BCG infection, mice were infected with 1 × 10^7^ colony-forming units (CFUs) of WT, ΔTCC and *comp*, through the intraperitoneal injection. Four mice from each group were sacrificed after 21-day post-infection, and the spleen homogenates [prepared by homogenizing aseptically removed organs in 1 ml of sterile saline with Tissue-Tearor (Biospec Products) at a maximum of 2,500 g] were plated on 7H10 agar supplemented with 10% OADC, in triplicates. CFUs of *M. bovis* were counted after 4 weeks of incubation at 37°C.

### Flow Cytometry

Spleens of infected C57BL/6 mice were collected at day 21. Single-cell suspensions were generated from spleens in Dulbecco’s Modified Eagle Medium (DMEM) containing 10% fetal calf serum (FCS) and Penicillin-Streptomycin, red blood cells were lysed with red blood cell lysis buffer (BioLegend) followed by washing step. Cells were resuspended in FACS buffer and incubated with the Live/Dead marker DRAQ7 (BioLegend) for 5 min at 4°C in the dark. Cells were resuspended in FACS buffer and flow cytometry was performed to quantify cell populations. Details of antibodies used are as follows: CD4 phycoerythrin (PE)-Cy7 (BioLegend; clone RM4-5), CD8 V500 (BD Horizon, clone 53-6.7), CD62 allophycocyanin (APC) (BD Pharmingen; clone MEL-14), CD44 Pacific Blue (BioLegend, clone IM7), CD25-APC (eBioscience, clone PC61.5), Foxp3-eFluor450 (eBioscience, clone FJK-16S). Central memory CD4^+^ T cells were CD3^+^ CD4^+^ CD44^high^ CD62^high^, effector memory CD4^+^ T cells were CD3^+^ CD4^+^ CD44^high^ CD62L^low^, regulatory T cells were CD4^+^ CD25^+^ FoxP3^+^. To analyze CD4, CD25 and FoxP3 expression, cells were stained with anti-CD4, anti-CD25); then followed by intracellular staining with anti-FoxP3. Isotype-matched antibodies (eBioscience) were used as controls. After staining, the cells were analyzed using a BD FACS Aria II (BD Biosciences) and analyzed using FlowJo software (version 8.6.3). The gating strategy has been described in Li et al. (2021). The CD4^+^ CD25^+^ lymphocyte population was gated for FoxP3 expression analysis. The proportion of FoxP3^+^ cells in CD4^+^ CD25^+^ T cells were compared among the different groups.

### Immunohistochemical Analysis

Spleen specimens were fixed in 4% PFA (paraformaldehyde) and embedded in paraffin. Tissue blocks were sliced into 5 μm sections, air dried and fixed with cold acetone. Tissue slides were stained with primary antibodies against Foxp3 (CST, clone D6O8R, 1:800) for overnight incubation at 4°C. After incubation with primary antibody, staining was followed by ABC detection kit (Abcam) using biotinylated anti-rabbit IgG. AEC was used as chromogen. After counterstaining with hematoxylin, sections were dehydrated, mounted and examined under the Olympus BX63 microscope (Olympus). Specimens were scored according to the intensity of the dye color and the number of positive cells. The intensity of the dye color was graded as 0 (no color), 1 (light yellow), 2 (light brown), or 3 (brown), and the number of positive cells was graded as 0 (<5%), 1 (5–25%), 2 (25–50%), 3 (51–75%), or 4 (>75%). The two grades were added together and specimens were assigned to one of 4 levels: 0–1 score (−), 2 scores (+), 3–4 scores (++), more than 5 scores (+++). The positive expression rate was expressed as the percent of the addition of (++) and (+++) to the total number.

### Statistical Analysis

Statistical analysis was performed using Prism (version 7.0c; GraphPad Software). Statistical significance was assessed using Welch’s *t*-tests, unpaired *t*-tests, one-way ANOVA followed by Dunnett’s multiple comparison test, or two-way ANOVA followed by Turkey’s multiple comparisons test wherever appropriate. The statistical tests used, exact *p*-values, and sample sizes are indicated in each figure legend. A *p*-value of 0.05 or less was considered statistically significant.

## Results

### Type III-A CRISPR-Cas System Is Required for Adaptation to Oxidative Stress

**FIGURE 1:**
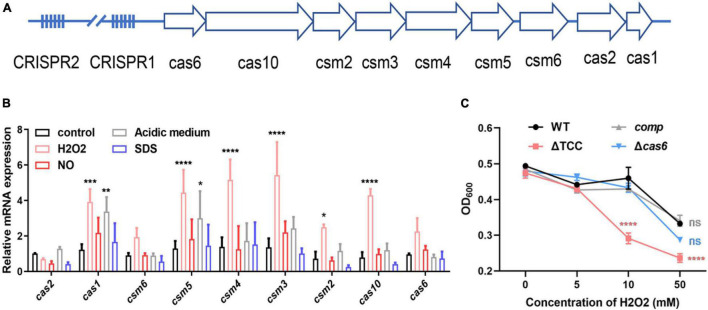
Inactivation of type III-A CRISPR-Cas system decreased bacterial survivability under oxidative stress. **(A)** Schematic of the *Mycobacterium bovis* BCG type III-A CRISPR-Cas operon. The type III-A CRISPR-Cas loci comprise *csm2-6*, *cas10*, *cas6*, *cas1*, *cas2*, and two CRISPR locus. **(B)** Fold changes in mRNA level of nine *cas* genes during different stresses. BCG was grown in 7H9 medium containing OADC and Tween 80 to mid-log phase. These strains were treated with different stresses, including 5mM NO, acidic medium (pH4.5), 1% SDS, 5mM H_2_O_2_. The expression of *cas* genes was analyzed by real time PCR (*n* = 3 biological replicates per group, data are shown as the mean ± SEM, two-way ANOVA with Tukey’s multiple comparison test, compared with control group; *p < 0.05, **p < 0.01, ****p < 0.0001). **(C)** Differential susceptibility of WT BCG, ΔTCC, *comp*, or Δ*cas6* strains to H_2_O_2_ compared to control was determined by OD_600_ measurements. *M. bovi*s BCG were grown in 7H9 medium containing OADC and Tween 80 to mid-log phase. The cultures were treated with different amounts of H_2_O_2_. OD_600_ were recorded (*n* = 3 biological replicates per group, data are shown as the mean ± SEM, two-way ANOVA with Tukey’s multiple comparison test, compared with WT group; ns, not significant, ***p < 0.001, ****p < 0.0001).

*Mycobacterium bovis* BCG CRISPR-Cas locus possesses two CRISPR regions and nine *cas* genes (Figure 1A and Supplementary Figure 1), similar to *M. tuberculosis* type III-A CRISPR-Cas system. Since *M. tuberculosis* encounters different degrees of adverse niche, such as the acidic, nitro-oxidative stresses, when growing in the intracellular environment, we first measured transcription level of nine cas genes in *M. bovis* BCG under such stresses. The mRNA levels of *cas1*, *csm5*, *csm4*, *csm3*, *csm2*, and *cas10* were significantly increased in response to oxidative stress (Figure 1B), and the expression of *csm6* was increased but without significance. In addition, only *cas1* and *csm5* was significantly upregulated upon acidic medium treatment. CRISPR-associated genes were not induced after NO or SDS exposure. These results indicated that potential role of type III-A CRISPR-Cas in resistance to oxidative stress. To further explore the function of type III-A CRISPR-Cas system in mycobacteria, two mutant strains, Δ*cas6* and ΔTCC (*t*otal III-A *C*RISP*R*-Cas, including *cas6*, *cas10*, and *csm2*-*6*), were generated in *M. bovis* BCG by specialized transducing mycobacteriophages (Supplementary Figure 2). To confirm the phenotype caused by deletion of TCC, the complement strain (*comp*) was constructed by expression of TCC on the chromosome. The strains of WT, ΔTCC, *comp*, and Δ*cas6* were treated with hydrogen peroxide. Consistently, deletion of TCC conferred reduced survivability after exposure to hydrogen peroxide compared to parental bacteria and complement strain (Figure 1C). In addition, the percentage growth of Δ*cas6* mutant strain was significantly reduced compared to WT strain upon the treatment with 50 mM. These results indicated that type III-A CRISPR-Cas system contributed to oxidative stress resistance.

### TCC Enhances Mycobacterial Envelope Integrity

**FIGURE 2:**
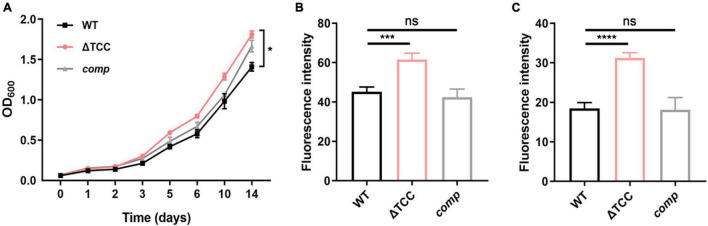
The effect of TCC deletion on mycobacterial cell wall permeability. **(A)** WT, TCC deletion mutant, *comp* strains were grown to mid-log phase, washed. Bacteria were diluted to an initial OD_600_ of 0.05 and the OD_600_ was monitored at the designated time points. Cell growth was monitored by OD_600_ measurements (*n* = 3 biological replicates per group, data are shown as the mean ± SEM, two-way ANOVA with Tukey’s multiple comparison test, compared with WT group; *p < 0.05). **(B,C)** WT, TCC deletion mutant, *comp* strains were grown to mid-log phase, washed, and stained with **(B)** Nile red or **(C)** ethidium bromide, and fluorescence was measured (*n* = 3 biological replicates per group, data are shown as the mean ± SEM, one-way ANOVA with Dunnett’s multiple comparison test, compared with WT group; ns, not significant; ***p < 0.001, ****p < 0.0001).

Next, we sought to explore the phenotype caused by TCC mutant. It has been previously demonstrated that *cas* genes deletion impacts bacterial biofilm formation (Cui et al., 2020). To examine whether type III-A CRISPR-Cas system can regulate biofilm formation, WT, ΔTCC, and *comp* strains were cultured in Sauton’s medium. Compared to WT and *comp* strains, no significant biofilm change was observed in ΔTCC (Supplementary Figure 3). Interestingly, the colony of ΔTCC was rougher than WT and complement strain. In liquid media, ΔTCC grew slightly faster with similar growth curves (Figure 2A). In light of the observation of abnormal morphology due to loss of TCC, we speculated whether TCC enhanced mycobacterial envelope integrity. To explore this, the permeability of ΔTCC was analyzed by measuring its uptake of Nile red. TCC deletion mutant exhibited a significant increase in fluorescence intensity compared with WT bacteria, indicating that it is more permeable to Nile red (Figure 2B). Furthermore, similar experiments were performed with the nucleic acid-staining dye ethidium bromide (Figure 2C) and a significant increase in fluorescence intensity was observed in the TCC mutant. Thus, our results demonstrated that TCC contributed to mycobacterial envelope integrity.

### Analysis of Transcriptome Profile Alterations in ΔTCC

**FIGURE 3:**
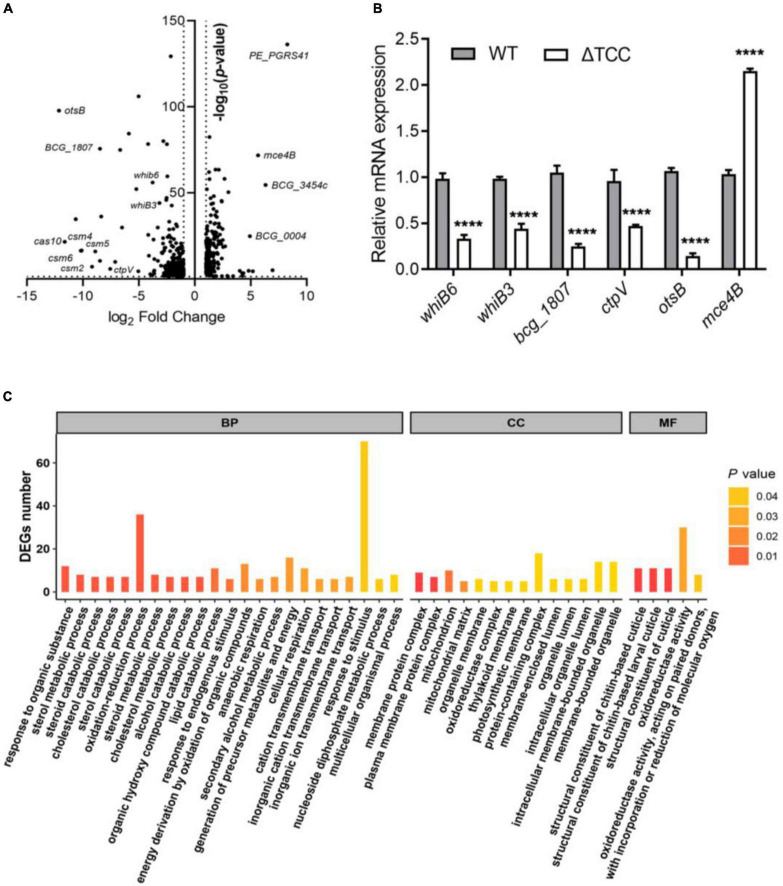
Gene-expression differences between WT and ΔTCC. **(A)** The volcanic diagram of gene expression difference between WT and ΔTCC strains determined by RNA-seq. The significantly upregulated and downregulated genes [*p* ≤ 0.05 and | log_2_ fold change (ΔTCC/WT) | ≥ 1] were shown. **(B)** The mRNA level of differential genes was tested by qRT-PCR. WT and ΔTCC strain were grown to log phase, the bacterial pellet was harvest for mRNA detection (*n* = 3 biological replicates per group, data are shown as the mean ± SEM, two-way ANOVA with Tukey’s multiple comparison test, compared with control group; ****p < 0.0001). **(C)** Gene ontology (GO) annotations of differentially expressed genes and classification graph of GO terms for differentially expressed genes. GO includes the molecular function (MF), cellular component (CC), and biological process (BP).

To understand the described phenotypes of the ΔTCC mutant, we next sought to identify the genes regulated by TCC. Although it has been reported that RNA targeting in type III-A CRISPR-Cas systems in *Thermus thermophilus* (Staals et al., 2014), no apparent homology was found between the genome of *M. bovis* BCG and spacer sequences in type III-A CRISPR locus. To shed light on the functional roles of TCC, we performed whole-transcriptome RNA-seq analyses to identify differentially expression genes in ΔTCC strains. Wild-type *M. bovis* BCG and ΔTCC were collected at log-phase (OD_600_, 0.5) for RNA-seq (see section “Materials and Methods”). With our significant criteria (|log_2_ fold change (ΔTCC/WT)| ≥ 1 and *p*-value < 0.05), 590 genes were identified as differentially expressed genes, 220 of which were upregulated and 370 were downregulated in ΔTCC. Differentially expressed genes were shown in the volcano plot (Figure 3A, and Supplementary Table 2). Of note, the positive control in our experiments, cas genes, were the most down-regulated genes in the entire dataset and 84 cell wall proteins were differential expressed (Supplementary Table 3). Consistently, mRNA expression of *whiB6*, *whiB3*, *bcg_1807*, *ctpV*, and *otsB* that downregulated in ΔTCC, significantly decreased as shown in Figure 3B. Also, *mce4B* belonging to cholesterol transport system mce4, was increased in ΔTCC. Moreover, differentially expressed genes were mapped to the KEGG database and enriched KEGG pathways were analyzed. The 62 enriched pathways (FDR > 0.05) are shown in Supplementary Table 4. In addition, to investigate the potential biological functions in which differentially expressed genes might be involved, the differentially expressed genes were enriched in GO (Gene Ontology). The top three categories were oxidation-reduction process (BP, 36 genes), response to stimulus (BP, 70 genes), and oxidoreductase activity (MF, 30 genes) (Figure 3C). Of note, pathways related to fatty acid metabolism were significantly enriched in CC, such as cholesterol catabolic process and lipid catabolic process, indicating the impact of TCC on lipid metabolism. All the enriched GO terms are presented in Supplementary Table 5. These outcomes demonstrated that inactivation of TCC has a profound effect on cellular gene regulation, especially on fatty acid metabolism.

### Knockdown of TCC-Regulated Genes Conferred Reduced Survivability Upon Oxidative Stress

**FIGURE 4:**
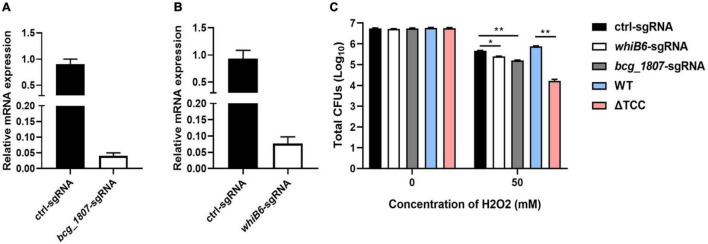
Knockdown of TCC-regulated genes decreased the resistance of BCG to hydrogen peroxide. **(A,B)** Using CRISPRi to target gene silence. Bacteria was collected at logarithmic phase in the presence of ATc. Gene knockdown was quantified by qRT-PCR (*n* = 3 biological replicates per each group, data are shown as the mean ± SEM). **(C)** Testing the survivability of knockdown strain. After induction, these knockdown strains were treated with 50 mM hydrogen peroxide. Bacteria strains were serially diluted 10-fold and spotted on 7H10-OADC agar. CFUs of *M. bovis* BCG were counted after 4 weeks days of incubation at 37°C (*n* = 3 biological replicates per group, data are shown as the mean ± SEM by unpaired *t*-test with two tailed *p*-value; *p < 0.05, **p < 0.01).

To determine whether disruption of TCC resulted in decreased survivability upon oxidative stress by regulation of gene expression, *whiB6* and *bcg_1807* were silenced by CRISPR interference (Rock et al., 2017), a robust tool for gene knockdown. WhiB6, a transcriptional regulator is important for *M. tuberculosis* virulence by modulating ESX-1 and the Dos regulon (Chen et al., 2016). BCG_1807 is an oxidoreductase which dysregulated response to stress (Leversen et al., 2012). We designed sgRNA to target *whiB6* and *bcg_1807* in BCG, respectively. After induction of CRISPRi, the knockdown efficiency was assessed by real-time PCR. The mRNA level of *whiB6* and *bcg_1807* was 10- and 20-fold lower than ctl-sgRNA, suggesting successful knockdown of target genes (Figures 4A,B). Next, knockdown strains were treated with H_2_O_2_. Consistent with our previous results, the survivability of ΔTCC was significantly decreased when compared to WT. Also, knockdown of *whiB6* showed slightly decreased survival, and the *bcg_1807* knockdown was significantly compromised for oxidative stress (Figure 4C), suggesting that downregulated *whiB6* and *bcg_1807* to some extent participated in ΔTCC-conferred reduced survivability in oxidative stress conditions.

### Intracellular Fitness of ΔTCC Mutant Strain Was Evaluated *in vivo* and *in vitro*

**FIGURE 5:**
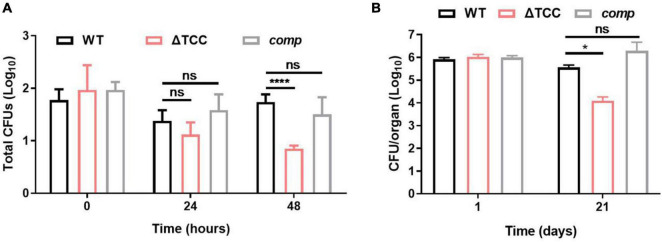
Evaluation intracellular fitness of ΔTCC mutant strain *in vivo* and *in vitro*. **(A)** RAW264.7 cells were seeded at 3 × 10^5^ cells per well in 24-well culture plates. After adhesion, cells were infected with bacteria at MOI of 10. The extracellular bacteria were removed 2 h post infection by washing with PBS. The cells of *M. bovis* BCG were further incubated for 24 and 48h at 37°C. Then serially diluted (10-fold) cells lysates were spotted on a 7H10-OADC agar and cultured in 37°C. CFUs were counted after incubation for the indicated time (*n* = 3 biological replicates per group, data are shown as the mean ± SEM, two-way ANOVA with Tukey’s multiple comparison test, compared with WT group; ns, not significant, ****p < 0.0001). **(B)** Mice were infected i.p. (1 × 10^7^) with WT, ΔTCC, or *comp* strains of *M. bovis* BCG. The number of surviving bacilli by CFU counting at the indicated time (*n* = 4 biological replicates per group, data are shown as the mean ± SEM, two-way ANOVA with Tukey’s multiple comparison test, compared with WT group; ns, not significant, *p < 0.05).

*Mycobacterium tuberculosis* use macrophages to traverse host epithelial barriers to enter deeper tissues, where they recruit additional macrophages to form granulomas. *M. tuberculosis*-infected macrophages generate reactive oxygen species (ROS) such as hydrogen peroxides and nitric oxide to control mycobacterial growth in the hyperinflammatory environment of the granuloma (Dallenga et al., 2017). Given that type III-A CRISPR-Cas system is required for resistance to oxidative stress which mimicks intracellular niche, we postulated that TCC mutant might exhibit reduced bacterial survivability *in vitro* and *in vivo*. To test this, RAW264.7 were infected with WT, ΔTCC, or *comp* strains and the numbers of surviving intracellular bacteria were assessed at 0, 24, and 48 h after infection. We found deletion of TCC significantly reduced the number of surviving intracellular mycobacteria at 48 h (Figure 5A). In addition, the expression of cytokines was examined following macrophage infection and we found that IL-10 mRNA expression was slightly increased in the macrophage cell line RAW264.7 infected with ΔTCC compared to WT or *comp* strains (Supplementary Figure 4).

Next, survival of WT, ΔTCC, and *comp* were assessed by CFU in mice. At 1-day postinfection there were no differences in the number of bacteria from the spleen. After 21-day of infection, we observed a decrease of bacterial burden in mice infected with the TCC mutant strain (Figure 5B). Together, these data suggested the essential role of type III-A CRISPR-Cas system in intracellular mycobacterial survival.

### Deletion of TCC in *M. bovis* BCG Is Associated With Increased Regulatory T Cell Population in Animal Model of Infection

**FIGURE 6:**
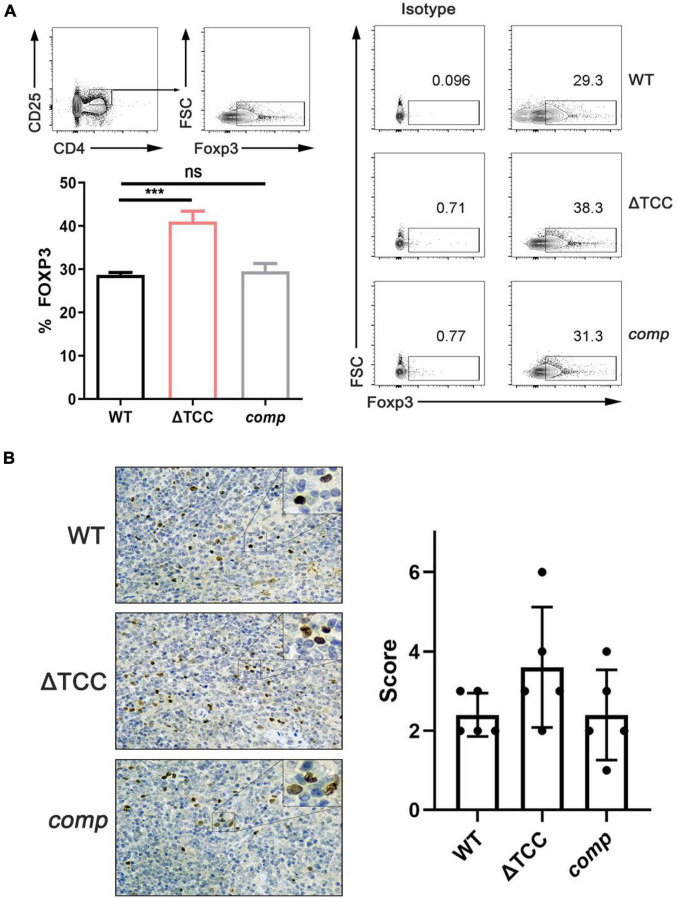
TCC is required for inhibition of regulatory T cells. **(A)** Flow cytometry evaluation of CD4^+^ CD25^+^ FOXP3^+^ cells in spleens from mycobacteria-infected mice. FOXP3^+^ cells were determined by intracellular staining with anti-FOXP3 after surface staining with anti-CD4 and anti-CD25^+^. Cells were stained with FOXP3 as a marker for regulatory T cells. Isotypic antibodies were used as controls. The proportion of FoxP3^+^ T cells within total CD4^+^ CD25^+^ T cells was significantly higher in ΔTCC compared to WT (*n* = 3 biological replicates per each group, data are shown as the mean ± SEM, one-way ANOVA with Dunnett’s multiple comparison test performed for ΔTCC, compared with WT group; ns, not significant, ***p < 0.001). **(B)** Immunohistochemical evaluation of FOXP3^+^ cells in mice spleens. 400× magnifications, respectively. Similar results were observed in all spleens evaluated (*n* = 5). Bar = 200 μm. Specimens were scored according to the intensity of the dye color and the number of positive cells.

It is known that the type I and type II CRISPR-Cas systems contribute to dampen the host immune response (Sampson et al., 2013; Li et al., 2016), but the role of type III CRISPR-Cas system in host immune response is unclear. *M. tuberculosis* possesses an unusual cell wall dominated by lipids and carbohydrates that is crucial for its survival and virulence. For example, mannose-capped lipoarabinomannan, a mycobacterial wall component, enabled expansion of regulatory T cell. Given that altered colony morphology and changing in transcript of cell wall component caused by TCC mutant, we hypothesized that type III CRISPR-Cas system may be involved in regulating host immune response. To verify our hypothesis, mice were infected with *M. bovis* BCG, ΔTCC, or *comp* strains. At day 21, no significant effect was observed in frequencies of central memory and effector memory CD8^+^ T cells, central memory CD4^+^ T, and effector memory CD4^+^ T cells between WT and ΔTCC (Supplementary Figure 5). Interestingly, frequencies were significantly increased for CD4^+^ CD25^+^ Foxp3^+^ T cells in the spleen of mice infected with ΔTCC (Figure 6A). Moreover, increasing Foxp3^+^ cells in the spleen of mice infected with ΔTCC was verified by immunohistochemical analysis (Figure 6B), which is in line with our previous observation. Collectively, these observations support the conclusion that TCC contributed to reduced CD4^+^ CD25^+^ Foxp3^+^ regulatory T cell population in BCG infected mice.

## Discussion

As a remarkably successful pathogen, *M. tuberculosis* is capable of persisting in host tissues for decades. Accumulating evidence described that *M. tuberculosis* employed several virulence factors to evade host immunity (Conrad et al., 2017; Wang et al., 2017; Sanchez et al., 2020). Type III-A CRISPR-Cas system was exclusively found in pathogenic mycobacteria, suggesting its unique role of type III-A CRISPR-Cas system in mycobacterial pathogenicity. In this study, we preliminarily described the important role of type III-A CRISPR-Cas system in mycobacteria. We found that the type III-A CRISPR-Cas system of *M. bovis* BCG is required for adaptation to oxidative stress and bacterial envelope integrity. Moreover, we found that TCC deletion increases the sensitivity of BCG to H2O2 through modulation of cell wall component. Consistent with the above findings, TCC deficiency attenuates the intracellular survivability, suggesting that the type III-A CRISPR-Cas system may be a novel target for therapy. Furthermore, we demonstrated *in vivo* that TCC contributes to regulate the regulatory T cell population during infection *in vivo*.

It has been described that the expression of CRISPR-Cas system was controlled by quorum sensing signaling molecules in *P. aeruginosa* and *Chromobacterium violaceum* (Patterson et al., 2016). Unlike *P. aeruginosa*, the LuxS synthase that is responsible for synthesis the quorum sensing signaling molecules autoinducer-2 is absent in *M. tuberculosis*. Therefore, autoinducer-2 failed to act as an autoinducer in *M. avium* (Geier et al., 2008). A recent study demonstrated that control of the CRISPR-Cas system by CnpB may be mediated through nanoRNAs (2–5 nucleotides), instead of hydrolysis of c-di-AMP or c-di-GMP, indicating that the accumulation of nanoRNAs may initiate the mRNA transcript of cas genes. Indeed, various stresses, such as low pH and hydron peroxide, to some extent, contributes to nanoRNAs accumulation (Nickels, 2012). Intriguingly, we found that cas genes were upregulated after H_2_O_2_ treatment, but not after NO treatment. It appears *M. tuberculosis* exhibited different transcriptional response after H_2_O_2_ and NO, respectively. For instance, *M. tuberculosis* exposed to 5 mM H_2_O_2_ resulted in massive transcriptional changes without an effect on growth or survival. Unlike H_2_O_2_ exposure, 5 mM NO exposure caused bacteriostatic activity. Moreover, after exposure to the same concentration of H_2_O_2_ and NO, a similar but different transcriptional response of *M. tuberculosis* was observed. After H_2_O_2_ exposure, DNA damage response and several PE and PPE genes were differentially expression that was not observed upon NO treatment (Voskuil et al., 2011).

Our data also demonstrated that inactivation of TCC resulted in reduced resistance to hydrogen peroxide. There are two possible explanations of increased sensitivity of ΔTCC to hydrogen peroxide. First, deletion of TCC significantly increased the envelope permeability. Increased cell-envelope permeability may lead to the enhanced uptake of compounds (Arts et al., 2015; Dulberger et al., 2020), such as hydron peroxide. Second, TCC influenced oxidation-reduction process. Indeed, in comparison to WT strain, several known transcriptional regulators that linked to oxidative stress, were significantly downregulated in ΔTCC (Figure 3A and Supplementary Table 2). For example, WhiB3 (BCG_3486) as an intracellular redox sensor maintained redox homeostasis (Saini et al., 2016). Deletion of *whiB3* resulted in reduced viability in response to H2O2 and NO (Mehta and Singh, 2019). A RNA polymerase sigma factor SigE was induced upon oxidative stress (Jensen-Cain and Quinn, 2001) and *sigE* mutant was more sensitive to oxidative stress than its parent strain (Manganelli et al., 2001).

Accumulating evidence demonstrated that CRISPR-mediate transcriptional interference of the endogenous target could facilitate bacterial pathogenesis (Sampson et al., 2013; Li et al., 2016; Ratner et al., 2019). Although no apparent homology was found between the genome of *M. bovis* BCG and spacer sequences in CRISPR loci, whole-transcriptome RNA-seq identified 590 differentially expressed genes in TCC deletion strains, of which the majority were associated with metabolism, suggesting the profound effect of type III-A CRISPR-Cas system on mycobacteria viability. Of note, several virulence associated genes were significantly downregulated in ΔTCC. For example, CtpV is a metal cation-transporting ATPase and mutant for *ctpV* attenuated bacterial survival *in vivo* and *in vitro* (Ward et al., 2010). *otsB* is a gene involved in trehalose biosynthesis and the *otsB* deficiency in *M. tuberculosis* led to intracellular accumulation of trehalose-6-phosphate and therefore resulted in reduced bacterial survival in mice (Vanaporn and Titball, 2020). In addition, transcriptional regulator WhiB6 (BCG_3925c), was significantly down-regulated in TCC mutant strain, it raised the possibility that type III-A CRISPR-Cas system may involve in the regulation of bacterial virulence due to WhiB6-mediated control of type VII secretion system ESX-1 modulated mycobacteria virulence (Chen et al., 2016; Bosserman et al., 2017). This suggested that type III-A CRISPR-Cas system of BCG may be involved in bacterial virulence through WhiB6-mediated regulation of type VII secretion system ESX-1, indicating importance of type III-A CRISPR-Cas system in mycobacterial survival *in vivo*. Because type VII secretion system ESX-1 is absent in the avirulent strain *M. bovis* BCG, it would be interesting in future studies to examine the role of type III-A CRISPR-Cas system in the virulence of pathogenic *M. tuberculosis*.

There are a few caveats of our study that are worth discussing. First, we observed that TCC genes were significantly induced upon oxidative stress, and disruption of TCC resulted in reduced viability after hydrogen peroxide treatment. Our data may give rise to three independent lines of evidence in support of our hypothesis that TCC maintained the envelope integrity. (i) ΔTCC exhibited a distinct colony morphology compared to WT and complement strain (Supplementary Figure 3). Actually, abnormal morphology is associated with the alteration of cell wall components (Bhatt et al., 2007; Reddy et al., 2013; Singh et al., 2016); (ii) TCC deletion mutant exhibited a significant increase in fluorescence intensity compared with WT bacteria (Figures 2B,C); (iii) Analysis of GO terms revealed that pathways related to fatty acid metabolism were significantly enriched in CC (Figure 3C), such as cholesterol catabolic process and lipid catabolic process. Moreover, in KEGG pathway, fatty acid degradation (Down_*P*-value = 0.0019, and Down_FDR = 0.058) and steroid degradation (Down_*P*-value = 0.048, and Down_FDR = 0.57) were enriched (Supplementary Table 4), indicating the impact of TCC on lipid metabolism. It is well known that *M. tuberculosis* possesses complex lipids on the cell wall surface that are critical for the integrity of mycobacterial cell envelope (Dulberger et al., 2020). Intriguingly, 84 cell wall proteins were differentialy expressed between ΔTCC and WT (Supplementary Table 3). Among these genes, *mce4B*, *mce4C*, and *mce4F* that belong to mce4 operon were upregulated due to loss of TCC. It is generally accepted that host lipids are the primary carbon source for *M. tuberculosis in vivo*. Cholesterol transport system mce4 was essential for growth with macrophage (Pandey and Sassetti, 2008). The *mce4* mutants unable to metabolize cholesterol also showed important defects in intracellular growth or survival (Griffin et al., 2012). Together, these results are consistent with the possibility that type III-A CRISPR-Cas system is involved in the biosynthesis of cell wall lipids. Exploring these interactions would be an interesting future research direction. Second, ΔTCC mutant exhibited a reduced intracellular fitness *in vivo* and we only tested the inflammatory response in BCG-infected macrophage. It is unknown whether other immune cells, such as Th1 and Th17 cells, may involve in reduced survivability caused by TCC deletion. It is well-established that Th1 and Th17 cells are needed in order to control infection by *M. tuberculosis* (Cardona and Cardona, 2019). Mycobacterial cell envelope components, such as lipoarabinomannan (Sarmiento et al., 2019) and phosphatidyl-inositol mannosides (Toyonaga et al., 2016), were able to promote a Th1 response during infection. Investigating the impact of TCC on Th1 and Th17 response would be an interesting future research direction. Third, type III-A CRISPR-Cas system of BCG decreased the CD4^+^ CD25^+^ Foxp3^+^ T regulatory cell population. Although ΔTCC upregulated the expression of anti-inflammatory cytokine IL-10, a key cytokine involved in the immunosuppressive function of Tregs, the relationship between TCC and Treg cells remained unknown. Garg et al. demonstrated that treatment with mannose-capped lipoarabinomannan, a mycobacterial wall component, enabled expansion of regulatory T cell (Garg et al., 2008). It appeared that the expansion of regulatory T cell may link to ΔTCC-mediated cell wall modeling. In addition, there is a recurring debate as to whether Tregs are beneficial or detrimental in *M. tuberculosis* infection. For example, when CD25^+^ cells were depleted in DBA/2 mice prior to *M. tuberculosis* infection, a reduction in the bacterial load of lungs and spleen was observed (Ozeki et al., 2010). Depletion of CD25^+^ cells 3 days prior to infection in *M. bovis* BCG had no effect on bacterial load (Quinn et al., 2006). However, in another study, the adoptive transfer of *M. tuberculosis*-specific Treg before T cell expression results in higher bacterial load (Shafiani et al., 2010). Also, it is not yet clear whether high level of Tregs are a consequence of inflammation for development of tuberculosis (Cardona and Cardona, 2019). Thus, the detailed interesting mechanism by which type III-A CRISPR-Cas of *M. tuberculosis* impacts Treg expansion requires further investigation.

## Conclusion

Our study revealed the important role of type III-A CRISPR-Cas system in mycobacterial biology. The deletion of TCC resulted in decreased resistance to oxidative stress, damage of cell envelope, reduction of intracellular fitness, and increased T regulatory cells. The unappreciated role of type III-A CRISPR-Cas systems would help us to understand the pathogenesis of *M. tuberculosis*.

## Data Availability Statement

The datasets presented in this study can be found in online repositories. The names of the repository/repositories and accession number(s) can be found below: https://www.ncbi.nlm.nih.gov/geo/, GSE166137.

## Ethics Statement

All animal experiments were performed in accordance with the National Institutes of Health Guide for the Care and Use of Laboratory Animals, and the experimental procedures were approved by the Ethics Committee of Zhongshan School of Medicine on Laboratory Animal Care (reference number: 2016-159), Sun Yat-sen University. Written informed consent was obtained from the owners for the participation of their animals in this study.

## Author Contributions

SF: conceptualization. YL and MD: formal analysis. WL, LX, and DZ: investigation. FY, LL, and JL: methodology. G-BT: resources. SF, YC, and HZ: writing-original draft. DL: writing–review and editing. All authors contributed to the article and approved the submitted version.

## Conflict of Interest

The authors declare that the research was conducted in the absence of any commercial or financial relationships that could be construed as a potential conflict of interest.

## Publisher’s Note

All claims expressed in this article are solely those of the authors and do not necessarily represent those of their affiliated organizations, or those of the publisher, the editors and the reviewers. Any product that may be evaluated in this article, or claim that may be made by its manufacturer, is not guaranteed or endorsed by the publisher.

## Funding

This work has been supported by grants from National Natural Science Foundation of China (Grant Numbers 81722030, 81830103, 8201101256, and 82002173), the Qingyuan People’s Hospital Medical Scientific Research Fund Project (Grant Number 20190209), the Guangdong Provincial Bureau of Traditional Chinese Medicine research fund (Grant Number 20201407), National Key Research and Development Program (Grant Number 2017ZX10302301), Guangdong Natural Science Foundation (Grant Number 2017A030306012), project of high-level health teams of Zhuhai at 2018 (The Innovation Team for Antimicrobial Resistance and Clinical Infection), 111 Project (Grant Number B12003), and Open Project of Key Laboratory of Tropical Disease Control (Sun Yat-sen University), Ministry of Education (Grant Numbers 2020kfkt04 and 2020kfkt07).

## Supplementary Material

The Supplementary Material for this article can be found online at: https://www.frontiersin.org/articles/10.3389/fmicb.2021.774492/full#supplementary-material

## Footnotes

1
https://www.ncbi.nlm.nih.gov/genbank/

2
http://www.geneontology.org
