# Supplementary figures and images for: The Involvement of Mycobacterium Type III-A CRISPR-Cas System in Oxidative Stress
Source: Front Microbiol. 2021 Dec 9;12:774492. doi: 10.3389/fmicb.2021.774492 (PMC8696179; doi:10.3389/fmicb.2021.774492)

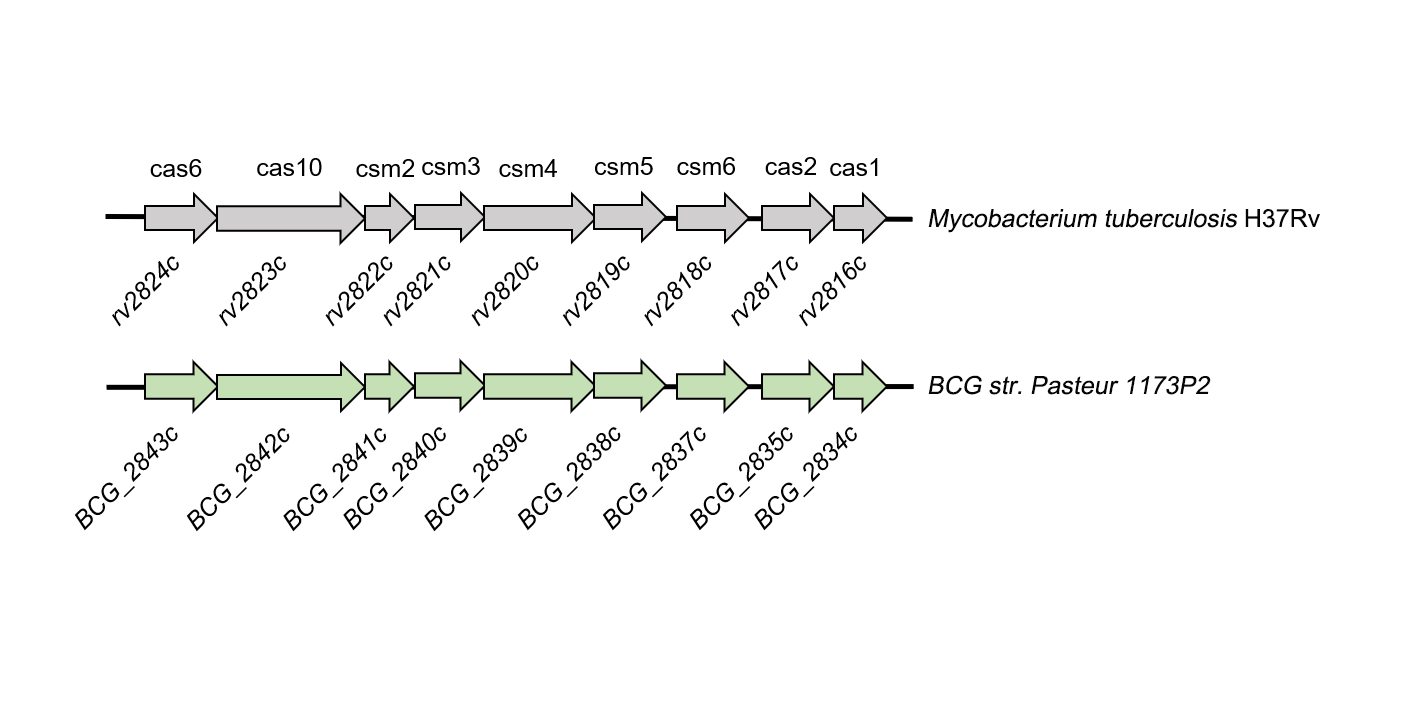

Supplement: Supplementary file 1 [file Image_1.tif]

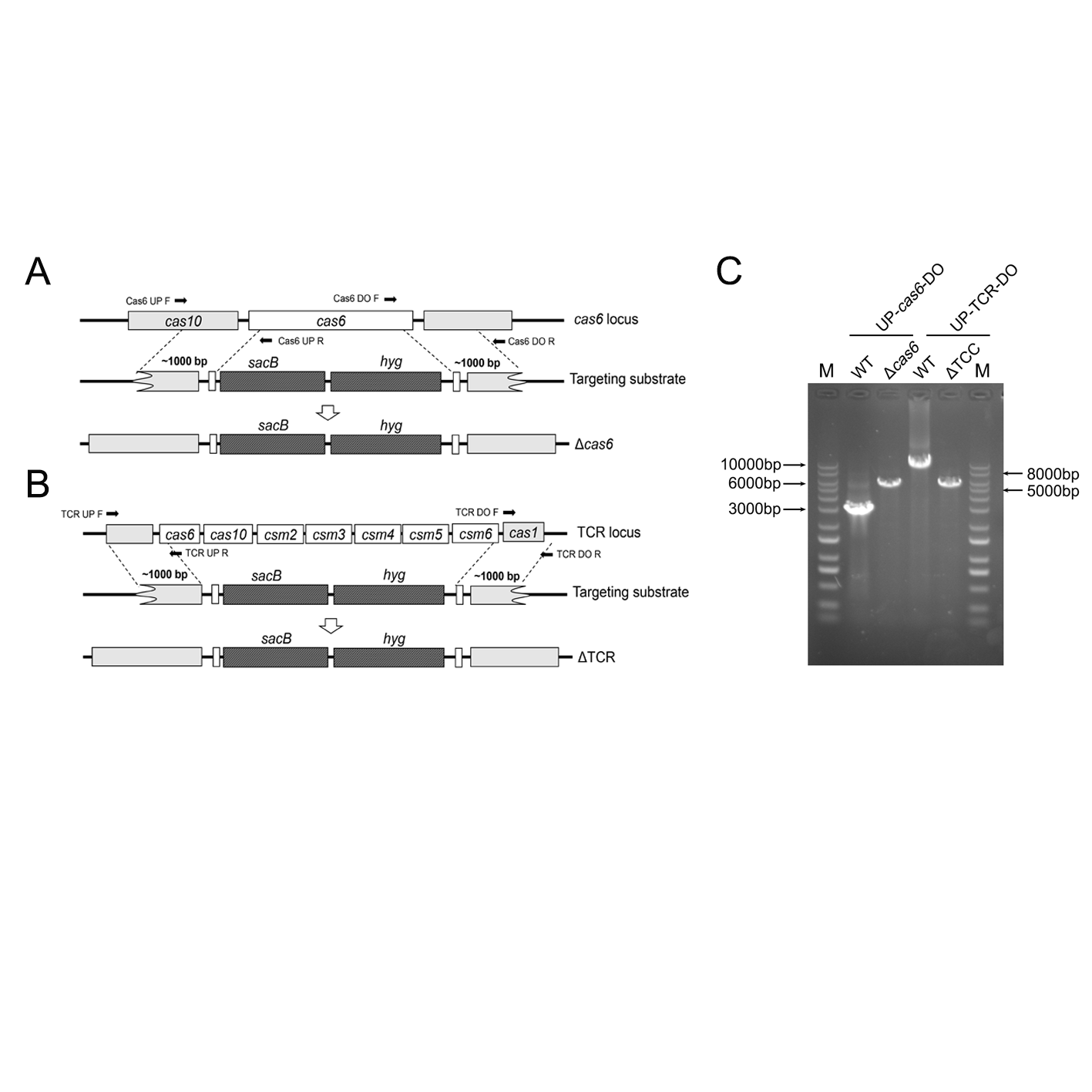

Supplement: Supplementary file 2 [file Image_2.tif]

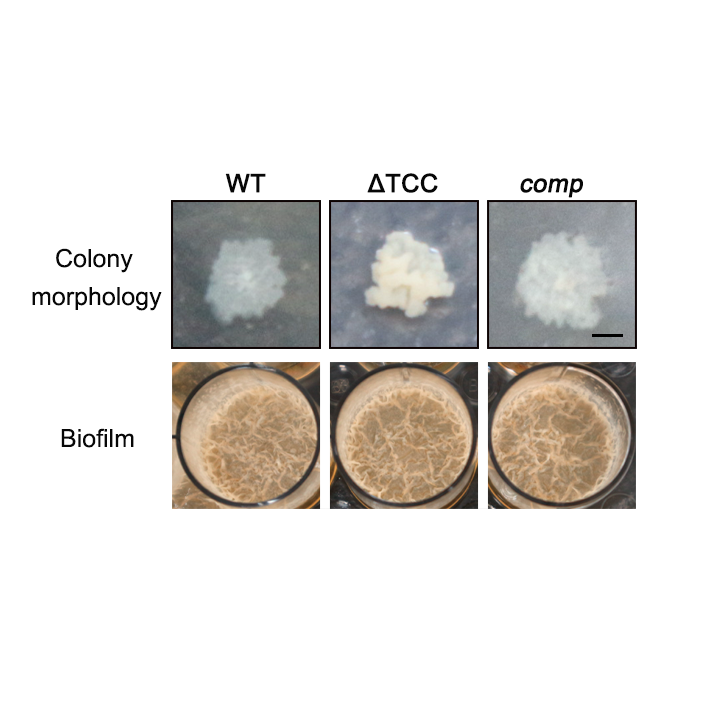

Supplement: Supplementary file 3 [file Image_3.tif]

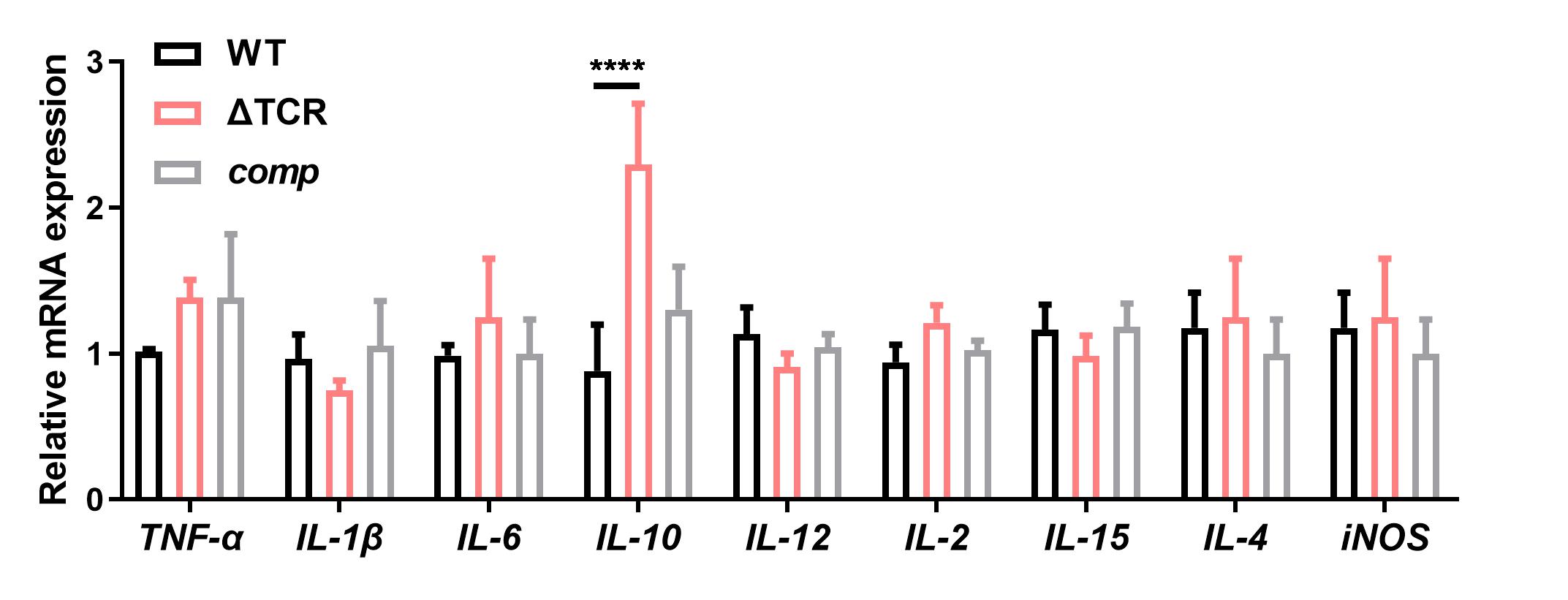

Supplement: Supplementary file 4 [file Image_4.jpg]

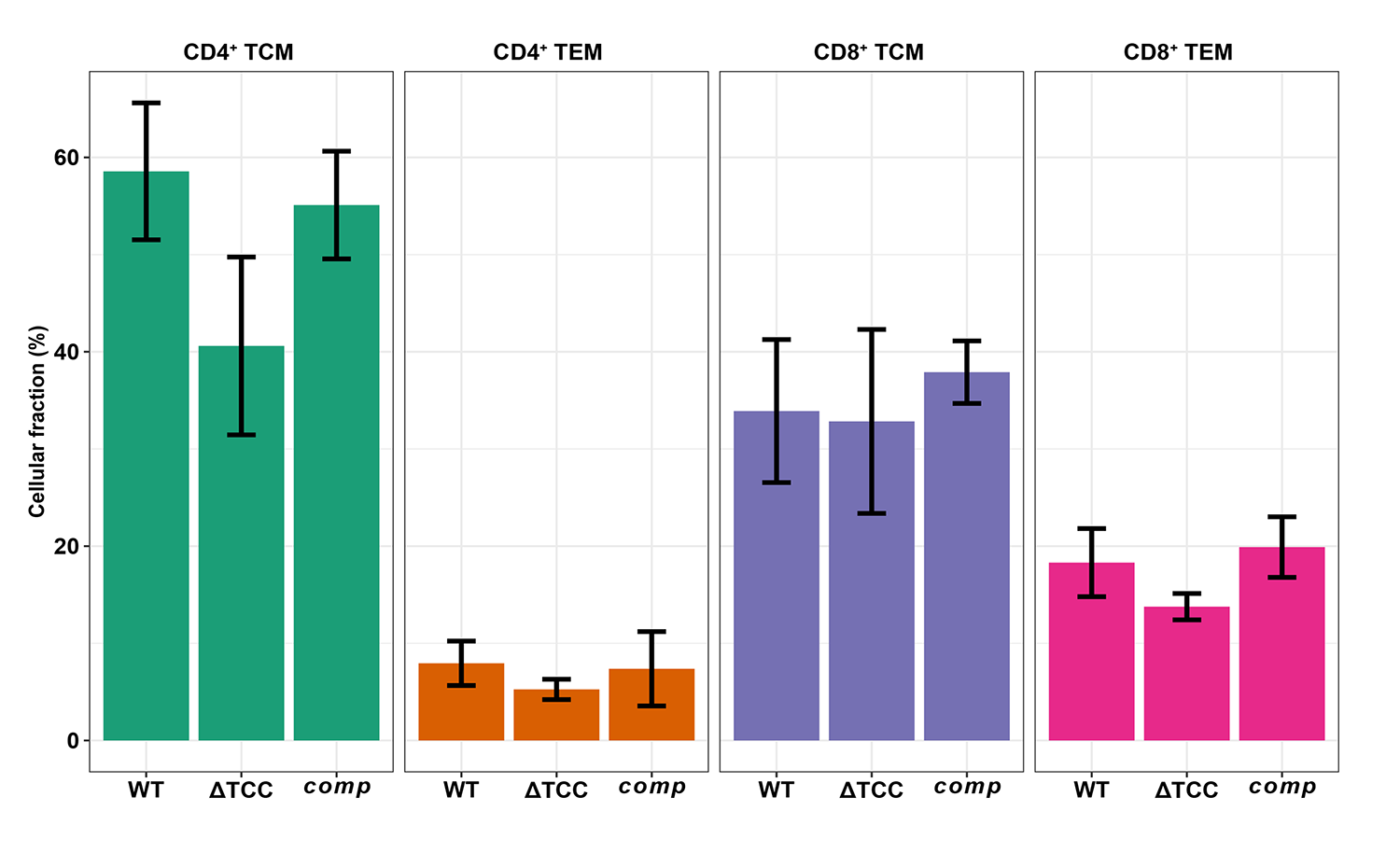

Supplement: Supplementary file 5 [file Image_5.tif]
